# Inflammation and Prostate Cancer: A Multidisciplinary Approach to Identifying Opportunities for Treatment and Prevention

**DOI:** 10.3390/cancers14061367

**Published:** 2022-03-08

**Authors:** Lanshan Huang, Melissa J. LaBonte, Stephanie G. Craig, Stephen P. Finn, Emma H. Allott

**Affiliations:** 1 Patrick G. Johnston Centre for Cancer Research, Queen’s University Belfast, Belfast BT9 7AE, UK; lhuang11@qub.ac.uk (L.H.); m.labontewilson@qub.ac.uk (M.J.L.); stephanie.craig@qub.ac.uk (S.G.C.); 2 Department of Pathology, The First Affiliated Hospital of Guangxi Medical University, Nanning 530021, China; 3 Department of Histopathology and Morbid Anatomy, Trinity Translational Medicine Institute, Trinity College Dublin, D08 HD53 Dublin, Ireland; stephen.finn@tcd.ie

**Keywords:** inflammation, immune, tumor microenvironment, prostate cancer, epidemiology, pathology, lifestyle

## Abstract

**Simple Summary:**

Prostatitis, or the inflammation of the prostate, is frequently observed in the clinic and by research studies, but its relevance to a man’s risk of being diagnosed with prostate cancer, or to his survival after the diagnosis, is not completely understood. In this review, we summarize the current knowledge on the causes of prostate inflammation, as well as the relationship with prostate cancer, with a particular focus on aggressive, defined as a high Gleason score or clinical stage, and lethal stages of disease. We also describe the strengths and weaknesses of the various technologies used to evaluate prostate inflammation in human studies, and we consider the potential of immune therapy and lifestyle interventions to prevent lethal disease and improve outcomes for prostate cancer patients. Future research is needed to better understand the role of prostate inflammation in lethal prostate cancer, and to provide evidence to guide the development of new treatment and prevention strategies to reduce prostate inflammation and improve survival.

**Abstract:**

Prostate cancer is a major cause of disease for men globally. Inflammation, an established hallmark of cancer, is frequently observed in the prostate, though its contribution to prostate cancer risks and outcomes is not fully understood. Prostate cancer is biologically and clinically heterogeneous, and there is now evidence that inflammation and immunological characteristics vary by the genomic and mutational landscape of the tumor. Moreover, it is now recognized that risk factor profiles vary between tumor subgroups, as defined by histopathological and molecular features. Here, we provide a review centered around the relationship between inflammation and prostate cancer, with a consideration of molecular tumor features and a particular focus on the advanced and lethal stages of disease. We summarize findings from epidemiological studies of the etiology and role of inflammation in prostate cancer. We discuss the pathology of prostate inflammation, and consider approaches for assessing the tumor immune microenvironment in epidemiological studies. We review emerging clinical therapies targeting immune biology within the context of prostate cancer. Finally, we consider potentially modifiable risk factors and corresponding lifestyle interventions that may affect prostate inflammation, impacting outcomes. These emerging insights will provide some hints for the development of treatment and prevention strategies for advanced and lethal prostate cancer.

## 1. Introduction

Prostate cancer is a major cause of disease among men, notable for its geographic variation, ranking the second most common men’s cancer for incidence and the fifth for mortality globally, with 1,414,259 new cases and 375,304 deaths estimated in 2020 [1]. Prostate cancer is biologically and clinically heterogeneous, and a major challenge lies in identifying, at the time of diagnosis, which cancers will be lethal. As such, there is an unmet need to understand the biology of lethal prostate cancer in order to inform prevention efforts and treatment strategies.

There is a large body of evidence supporting inflammation as a hallmark of cancer [2]. Inflammation is frequently observed in the prostate microenvironment, and has been hypothesized to be involved in prostate cancer initiation and progression [3]. Epidemiological studies assessing histological inflammation using hematoxylin and eosin (H&E)-stained tissues have reported both positive and inverse associations with prostate cancer risks [4,5,6] and outcomes [7,8,9,10]. However, the histological assessment of inflammation is a relatively crude measure, as inflammation is a complex and heterogeneous phenotype. Various technologies exist for determining the phenotype and activation status of immune cells, and their suitability for use in epidemiological studies depends on their compatibility with tissue preservation methods, the scalability of the technology for profiling sufficient numbers of individuals for a well-powered statistical analysis, and the desire to preserve spatial context. From a clinical perspective, it is intriguing that a relatively low proportion of patients achieve a response from currently available immunotherapies [11,12,13], suggesting that much of prostate cancer is likely to be immunologically “cold”. This could be characterized by lack of T-cells within tumor, or failed T-cell priming, such as ineffective antigen presentation [14]. Understanding the immune microenvironment across histological and molecular subgroups of prostate cancer may identify patients that could benefit from immunotherapy, as well as informing our understanding of lethal prostate cancer etiology to identify lifestyle interventions and prevention strategies.

In this review, we summarize the current knowledge on the relationship between inflammation and prostate cancer from the epidemiological perspective. We discuss the etiology and the role of inflammation in prostate cancer, with a particular focus on advanced and lethal disease, and we compare strategies for measuring inflammation in the context of epidemiological studies. Finally, we identify some future research directions, particularly for patient subgroups who may benefit from immune therapy and/or lifestyle interventions that target inflammation.

## 2. Epidemiological Associations between Inflammation and Prostate Cancer

Intraprostatic inflammation, or prostatitis, is clinically heterogeneous and comprises (I) acute bacterial prostatitis, (II) chronic bacterial prostatitis, (III) chronic prostatitis/chronic pelvic pain syndrome, and (IV) asymptomatic inflammatory prostatitis, according to the US National Institutes of Health (NIH) consensus [15]. Some have an infectious etiology and others have unknown causes, but all are characterized by prostate inflammation.

### 2.1. Infections in Prostate Cancer Etiology

An infectious etiology for prostate cancer has been put forward, supported by evidence from questionnaire-based and sero/urinary-epidemiological studies. The inflammatory response within the prostate could be induced by systemic dissemination or an organ-restricted infection. Sexually transmitted infections (STIs) have been studied as an etiologic factor. A meta-analysis, which included 47 studies published between 1971 and 2011, showed that men who reported a history of any STI had a 49% higher total prostate cancer risk [16].

Subgroup analyses within the above-mentioned meta-analysis showed a 20% higher total prostate cancer risk in those reporting a history of *Neisseria gonorrhoeae* [16]. Few studies have separated prostate cancer into histological subgroups. A population-based case-control study in Mexico reported higher odds of prostate cancer in men with a history of gonorrhea, relative to those without (OR 3.04; 95% CI 1.99–4.64), with similar estimates when stratified by the Gleason score, <7 vs. ≥7 [17]. An analysis within the prospective Health Professionals Follow-Up Study reported no association between gonorrhea and the total prostate cancer risk (RR 1.04; 95% CI 0.79–1.36), but found a suggestion of a positive association for advanced (stage 3b or higher) (RR 1.37; 95% CI 0.64–2.95), relative to organ-confined (RR 0.99; 95% CI 0.71–1.38), prostate cancer risk, although there were no differences in Gleason scores (<7 vs. ≥7) [18]. Therefore, while there is evidence supporting a higher risk of a total prostate cancer risk in men with a history of gonorrhoeae infection, additional studies that focus on advanced and lethal diseases are needed.

Two case-control studies nested within prospective cohorts examined the association of the *Trichomonas vaginalis* serostatus and prostate cancer risk, according to tumor features. A nested case-control study within the prospective Physicians’ Health Study reported that a *Trichomonas vaginalis* seropositivity was more strongly associated with an increased risk of advanced and lethal prostate cancer (OR 2.17; 95% CI 1.08–4.37 and OR 2.69; 95% CI 1.37–5.28, respectively) relative to the organ-confined disease (OR 1.10; 95% CI 0.81–1.49) [19], while a nested case-control study within the Health Professionals Follow-Up Study suggested a slightly stronger association with higher-grade tumors (Gleason score ≥ 7; OR 1.76; 95% CI 0.97–3.18 vs. <7; OR 1.27; 95% CI 0.79–2.06) [20]. However, a more recent analysis combining cases from these studies reported no association with prostate cancer-specific mortality [21]. A null overall effect (OR 0.97; 95% CI 0.70–1.34), and a suggestion of a lower risk for high-grade prostate cancer was reported, subsequently, within the Prostate Cancer Prevention Trial (PCPT), though this subgroup analysis was not statistically significant (OR 0.82; 95% CI 0.54–1.24) [22]. Other results from most recent studies showed that no significant associations existed between *Trichomonas vaginalis* and a prostate cancer risk, either overall, or stratified by tumor characteristics [23,24,25]. Together, these studies do not provide strong evidence for the role of *Trichomonas vaginalis* in prostate cancer.

Questions are also raised on whether mycoplasmas and viruses could play a role in the carcinogenesis and progression of prostate tumors. A meta-analysis of 10 studies showed that prostate cancer patients had 2.2-times higher odds of being colonized with any species of *Mycoplasma* spp. and 3.6-times increased odds with any species of *Ureaplasma* spp., relative to men with benign prostatic hypertrophy [26]. Results from a hospital-based study showed significantly higher serum concentrations of *M. hyorhinis* antibodies in prostate cancer patients with Gleason score of 7, relative to a Gleason score of 6 [27]. Two studies found evidence of *Ureaplasma* spp. in patients with more aggressive prostate tumors, by sequencing bacterial DNA present in urine samples [28] and prostate tissues [29]. These studies suggest a greater presence of mycoplasmas in aggressive prostate cancer, and future studies are needed to examine the relationship with advanced and lethal disease.

To date, strongly consistent evidence indicates that the BK virus (BKV) is a predisposing factor for different kinds of cancers, including prostate cancer [30,31], while other viruses, such as EBV, CMV, HSV2, HHV-8, XMRV, and HIV have inconsistent or null associations with prostate cancer. Mischitelli et al. [32] investigated the presence of BKV sequences in urine, blood, and fresh prostate cancer samples, utilizing a quantitative PCR assay. The number of viral copies decreased sequentially from the highest to lowest Gleason score, suggesting that BKV may play a role in the progression of prostate cancer, rather than its onset. A few serologic and molecular studies have observed that a human papillomavirus (HPV) infection tended to increase the risk of prostate cancer, but subsequent studies reported null or slightly inverse findings. Considering these studies together, an HPV-16 infection may represent a risk factor for total prostate cancer [33,34,35]. However, there is little evidence to support the hypothesis that HPV-16 or -18 infections were related to disease severity according to the Gleason score, the extent of the disease at diagnosis, or a combined measure of tumor aggressiveness [36].

In summary, genital infections with *Neisseria gonorrhoeae*, *Mycoplasma*/*Ureaplasma*, and BKV appear to be modestly associated with increased rates of prostate cancer. It should be addressed that we need to note the research methods and exposure assessment approaches when interpreting these results. If the epidemiological evidence arises from studies with a case-control design, the interpretation should take potential confounding into consideration, which can be caused by selection bias, recall bias, or detection bias. For example, men with STIs frequently show elevated serum prostate-specific antigen (PSA) levels compared to healthy controls [37], probably due to the damage of prostate epithelial cells and the release of PSA, extracellularly [38]. These men may, therefore, be more likely to undergo a prostate biopsy and be diagnosed with prostate cancer and, therefore, the detection bias could contribute to some of the observed associations between STIs and prostate cancer risk. However, this would not explain associations with the advanced or lethal stages of the disease. As mentioned above, epidemiological studies may use self-report methods or serology to assess STI exposure, namely infections caused by any of the STI agents, but these measures may not exactly represent the infection of the prostate by particular microorganisms. When considering studies using tissue analyses, we should be aware that the infections detected in the prostate cancer tissue may be acquired before, or after, the initiation of cancer. The consideration of these methodological challenges is needed when designing future studies to understand the role of infections by microbial species in aggressive, particularly lethal, prostate cancer, as well as the mechanisms contributing to these associations.

### 2.2. Histopathological Inflammation in Prostate Cancer

**Figure 1:**
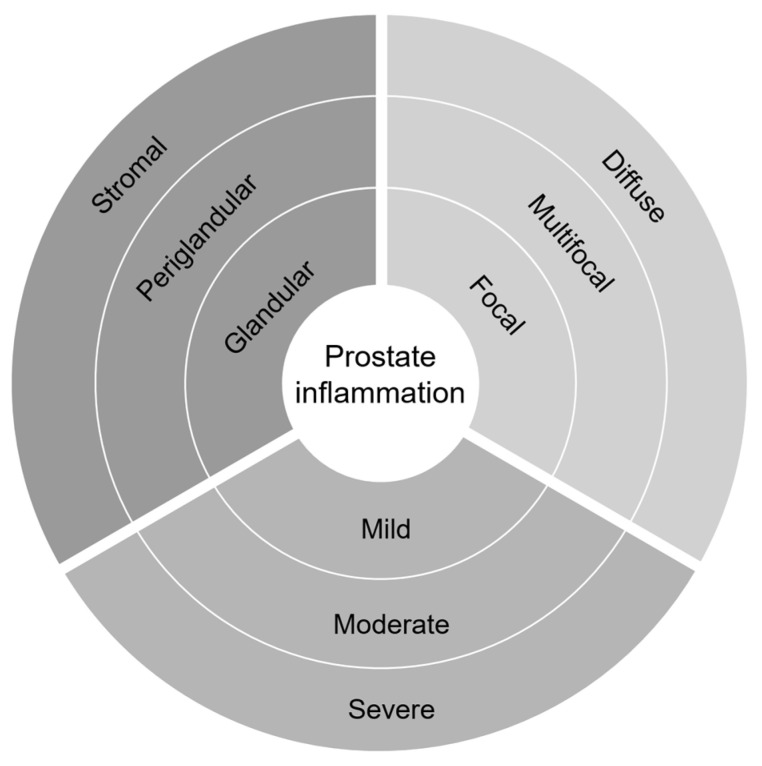
The histological classification of prostatic inflammatory infiltrates. Prostate inflammation is classified according to the inflammation extent (focal, ˂10%; multifocal, 10–50%; or diffuse, >50%) and grade (mild, ˂100 cells/mm^2^; moderate, 100–500 cells/mm^2^; or severe, >500 cells/mm^2^) in each tissue location (glandular, periglandular, and stromal). (Adapted from Nickel et al. [43]).

While the prostate may be exposed to a variety of microorganisms, inflammation in the prostate can also appear without a dominant infection and can present as histological prostatitis (i.e., asymptomatic prostatic inflammation). The cause of prostatic inflammation, in most cases, is indistinct, and it tends to be, incidentally, identified post-prostate biopsy or resection due to other certain prostate diseases, such as prostate cancer. Potential sources are postulated for the initial initiating event, including a direct infection as discussed above, chemical, and physical trauma induced by urine reflux, diet, estrogens, or a combination of two or more of these factors [39]. Different degrees of chronic or acute inflammation and inflammation-associated lesions are frequently observed in histological specimens of prostate tissue. The prevalence of chronic histological inflammation in prostate biopsies that are negative for cancer is high, reported at 78% in the PCPT [5], and 77% in the REduction by DUtasteride of PCa Events (REDUCE) trial [6]. It should be noted that there is an early histologic alteration induced by inflammation, called proliferative inflammatory atrophy, characterized by prostatic epithelia proliferation accompanied with atrophy, surrounded by inflammatory cell infiltration [40]. It shares many of the genomic and protein alterations that are exhibited by high grade-prostatic intraepithelial neoplasia and prostate cancer, such as *ERG* fusion and a lower expression of *NKX3.1*, strongly suggesting a potential preneoplastic transition and indicating an association between inflammation with the progression to prostate cancer [41,42]. A consensus of the histopathological classification system for prostatic inflammation, featuring the location of inflammatory infiltrates, as well as their extent and grade, has been proposed and applied in the cases using prostate biopsies, the transurethral resection of the prostate (TURP), or prostatectomy specimens (Figure 1) [43].

Studies assessing histological inflammation using H&E-stained tissues have reported contrasting associations with prostate cancer risk. Two studies by Platz et al. within the PCPT indicated that benign tissue inflammation was positively associated with the presence of prostate cancer, especially high-grade [4,5]. In their results, men with inflammation in benign regions of prostate biopsy tissue showed 1.78-times higher odds of being positive for prostate cancer, compared with cases without inflammation. The association was stronger for those with the more aggressive disease, defined as a Gleason score of 7–10, which had 2.24-times higher odds compared with cases with a lower Gleason score. In contrast, Moreira et al. found that the presence of acute and chronic inflammation in baseline negative prostate biopsies were both independently associated with lower odds of prostate cancer upon a subsequent biopsy in the REDUCE study [6]. To date, few studies have explored the association between histological inflammation at the time of prostate cancer diagnosis and prostate cancer outcomes [7,8,9,10]. An analysis of data from the Health Professionals Follow-Up Study showed an inverse association between the presence of histological inflammation in resected tumors, the adjacent normal prostate tissue, and the progression to lethal prostate cancer [10]. In contrast, a Swedish case-control study, where cases were early-stage prostate cancer patients receiving TURP, revealed a weak positive association between chronic histological inflammation and prostate cancer-specific mortality [8]. Klink et al. reported that prostate tumor inflammation was positively associated with biochemical recurrence in a subgroup of men treated with radical prostatectomy, but the association was no longer statistically significant after adjusting for pathologic features [9].

Overall, histological inflammation in prostate tissue is common, and its association with either the prostate cancer risks, or outcomes, could not be simply concluded based on current evidence. It is noteworthy that how the study population is selected might affect the findings. In countries with PSA screening, if men selected into the study were biopsied due to a high PSA and the cause of the elevated PSA was inflammation and not cancer, then the study may find that inflammation is inversely related to the presence of cancer, perhaps artificially. An explanation for these findings is the collider stratification bias, a form of selection bias occurring in studies where the study population is stratified or restricted by a collider—i.e., a shared effect of both the exposure and the outcome of interest. In this case, the PSA concentration is elevated in prostate inflammation as well as in prostate cancer [44]. More investigations of inflammation and prostate cancer risk, in the context of clinical studies with for-cause biopsies, will likely be subject to the same bias. Hence, it is worth considering other methods to assess prostate inflammation to reduce this issue. Non-invasive approaches, which do not require tissue sampling, may be good alternatives, and there is preliminary evidence that an imaging-based assessment of prostate inflammation may be feasible [45]. More investigations are still warranted to further clarify histological inflammation associations with prostate cancer risks and outcomes, particularly in the advanced/lethal disease.

### 2.3. Lifestyle Factors Altering Prostate Inflammation

Based on our understanding of inflammation and prostate cancer, interventions to control local and/or systemic inflammation to prevent prostate cancer seems reasonable and promising, which even would have the opportunity to reduce the progression and mortality of the disease (Table 1). Evidence from observational studies supports a potential role for physical activity, weight loss, dietary factors, estrogens, or a combination of two or more of these factors in affecting systemic and/or prostate inflammation [39]. For instance, a prospective study reported that vigorous exercise was associated with reduced risk of advanced and lethal prostate cancer and a lower risk of the *TMPRSS2:ERG* fusion-positive disease [46]. A gene set enrichment analysis of tumor-adjacent prostate tissue from this study suggested the altered expression of immune pathways in the tumor microenvironment of men who reported vigorous exercise, relative to those who did not [47]. An inflammatory dietary pattern was associated with an increased risk of lethal prostate cancer among younger men (<65 years of age) [48], and was predictive of higher serum concentrations of inflammatory biomarkers in men from the Prostate, Lung, Colorectal, and Ovarian cancer cohort [49]. In addition, serum fatty acid levels were associated with increased intraprostatic inflammation in the placebo arm of the PCPT [50], together suggesting that diet can alter both systemic and prostate inflammations. Cigarette smoking is also proposed to contribute to acute and chronic prostate inflammation [51] and has been associated with molecular alterations in prostate tissue characterized by an immune–inflammation signature [52]. Finally, anti-inflammatory agents, not only aspirin but also statins, have also shown their potential application in preventing prostate cancer progression. For example, regular aspirin use was associated with a lower risk of lethal prostate cancer, and post-diagnosis aspirin use was associated with reduced all-cause and prostate cancer-specific mortality [53]. Hurwitz et al. discovered low FoxP3 expression (a regulatory T-cell (Treg) marker) in aspirin users, and low CD68 expression (a macrophage marker) in statin users in benign prostate tissue from the placebo arm of the PCPT [54]. Lower levels of chronic and acute histological inflammation in prostate biopsies that were negative for prostate cancer were observed among statin users in the REDUCE study [55]. An analysis in the Health Professionals Follow-Up Study identified differential gene expressions of inflammation/immune pathways in tumor-adjacent prostate tissue as potential mechanisms linking statins with lower lethal prostate cancer risk [56]. Collectively, these observational findings support a role for a variety of potentially modifiable lifestyle factors in prostate inflammation, highlighting a potential contributing mechanism to their association with advanced and/or lethal prostate cancer.

## 3. Immunobiology in Prostate Cancer Microenvironment

The inflammation in prostate cancer tissues is mostly chronic, with a frequent observation of lymphocytes (i.e., tumor-infiltrating lymphocytes (TILs)), macrophages, and mast cells, whereas acute inflammation, comprised of neutrophils, is less common. In comparison with other cancer types, an understanding of the tumor immune microenvironment (TIME) in prostate cancer is still relatively poor, and prostate cancer is usually considered immunologically “cold” on the consensus of multiple trials, which means that this disease is poorly responsive to immunotherapy [11,12,13].

The classification of the immune status in primary prostate cancer is challenging for a number of reasons [3]. First, there are few morphologically apparent immune cells infiltrating the tumor in most prostate cancers and chronic inflammation is more common in benign regions. Second, it is challenging to determine the spatial distribution of immune cells in prostate cancer because of tumor multifocality and growth patterns, which are characterized by invasions between benign glands. Third, defining the tumor margin or peritumoral region in this disease is also difficult. As such, collaborative research efforts between molecular biology, epidemiology, and pathology disciplines will be key to understanding this biology.

### 3.1. Immune Modulators in Prostate Cancer Progression

Several advances in our understanding of the immune context of prostate cancer, TIME, have been achieved in recent years, including the immunological composition and function, spatial distribution, and heterogeneity. The immune modulators driving local cancer growth and distant dissemination consist of a broad spectrum of cells. A major factor determining cancer progression over time is the phenotype of T-cells within the TIME. Moreover, tumor-associated macrophages have been the most extensively studied and well-characterized [61]. Therefore, essential modulators in prostate cancer progression, including Tregs, Th17 cells, and M2 macrophages will be discussed here.

Tregs, a type of CD4+ T-cell in charge of inhibiting the activation and differentiation of CD4+ helper T-cells and CD8+ cytotoxic T-cells, have been identified as a suppressor of antitumor immune responses and play a role as a primary mediator in cancer progression. In a consecutive series of 102 men with prostate cancer undergoing radical prostatectomy, and 38 men without prostate cancer undergoing cystoprostatectomy for bladder cancer, those with epithelial CD4+ Tregs in benign prostatic tissue were four-fold more likely to have prostate cancer, and these tumors had a higher Gleason grade and stage [62]. In a study of men with prostate cancer classified either as indolent or lethal based on their survival over a 10-year follow-up period, the presence of each additional CD4+ Treg cell was associated with a 12% increase in the odds of lethal prostate cancer, independent of other clinical factors, such as the tumor stage, tumor volume, and Gleason score [63]. Another study suggested that the presence of C-C chemokine receptor 4 (CCR4) on tumor-infiltrating Tregs, which enabled the migration of Tregs into cancer tissues by chemotaxis, might indicate men who may progress to castration-resistant prostate cancer (CRPC) [64]. An advanced tumor stage was also positively associated with the number of CD4+ CD25+ FoxP3+ Tregs within the tumor [65,66].

Th17 cells, derived from CD4+ helper T-cells, are characterized by the generation of IL-17 [67]. The role of Th17 cells in cancer is controversial, and it is unclear whether the biological function of Th17 cells is pro- or anti-tumor in the context of prostate cancer. Previous studies showed that a dominance of Th17-mediated inflammation was linked to a lower tumor pathological grade in localized prostate cancer [68], and that the count of Th17 cells in peripheral blood inversely correlated with the time-to-disease progression in a small group of patients with hormone-refractory prostate cancer [69].

In addition to lymphocyte infiltration, high tumor-associated macrophage infiltration is pro-carcinogenic in the prostate cancer TIME, supported by both transcriptional landscape findings and differential tissue composition analyses, even spatial analyses, indicating that macrophage proximity to epithelial glandular clusters increased with tumor progression [70]. M2 macrophages are characterized by secreting high levels of M2-associated immunosuppressive cytokines and chemokines, among which TGF-β2 is the most highly expressed [71]. Men with abundant CD163+ M2 macrophages in prostate tissue have a higher risk of lethal prostate cancer and the interpretation is that the presence of these suppressor cells, CD163+ M2 macrophages, and CD4+ FoxP3+ Tregs may promote an immunosuppressive microenvironment [72]. The amount of immunosuppressive CD206+ M2 macrophages increased gradually from normal prostate tissue, to primary untreated cancers, to hormone-naïve regional lymph node metastases, to metastatic castration-resistant prostate cancer (mCRPC), potentially shedding light on the lack of clinical success of immunotherapy for prostate cancer patients [73].

Collectively, Tregs and M2 macrophages are functioning as promoters of prostate cancer progression, while Th17 cells might be involved in the anti-tumor process. Immune phenotypes of these modulators should always be considered in the immune context of high diversity and dynamic evolution.

### 3.2. Heterogeneity among Immune Phenotypes

There is heterogeneity in immunological composition, spatial distribution, and functions under diverse mutation-defined subtypes. The fusion of the androgen-regulated gene, *TMPRSS2*, with the oncogenic ETS transcription factor, *ERG*, is an early clonal event seen in roughly half of all prostate tumors [74]. Inflammation has been proposed as a potential mechanism driving *TMPRSS2:ERG* fusion by inducing oxidative stress which, in turn, can cause double stranded DNA breaks, thereby facilitating the formation of gene fusions [75]. Bacterial prostatitis has been associated with ERG-positive prostate cancer, providing evidence that infection-driven inflammation can initiate driver gene alterations to cause prostate cancer [41]. However, there are mixed findings regarding the presence of inflammation by the ERG-defined subtype. An inverse association between *TMPRSS2-ERG* fusion and TILs was identified by integrating an image analysis with RNA sequencing in 27 archival radical prostatectomy cases [76]. In contrast, Burdova et al. found that CD204+ macrophages and CD3+ T-lymphocytes may infiltrate the tumor region more intensely in *TMPRSS2-ERG* fusion-positive cases, compared to fusion-negative cases [77], in line with findings from Kaur and colleagues, showing increased T-cell density in ERG-positive tumors [78]. Aberrant levels of inflammatory mediators that changed with ERG expressions have also been discovered, and the imbalance of inflammatory mediators might impact the progression of ERG-positive prostate cancer with some loss of immune capability involving HLA-DMB molecule and CD3+ cells [79]. PTEN loss is observed more frequently in ERG-positive tumors, and PTEN-deficient prostate cancer has been shown to have an enhanced inflammatory infiltrate, with a greater density of T-cells [78] and a higher expression of immune-related genes [80]. PTEN-null prostate cancers have also been shown to present with an immunosuppressive tumor microenvironment characterized by an increased expression of IDO1 and a higher density of FoxP3+ Tregs in neoplastic glands, with distinct differences in infiltrating FoxP3+ Tregs or CD8+ T-cells at *PTEN*-deficient metastatic sites, such as bone, liver, and lymph nodes [81]. It is interesting that in *BRCA1/2* wild-type prostate cancer, immune cells are located predominantly extratumorally, whereas most *BRCA2*-mutated tumors show a significantly increased intratumoral immune cell infiltration [82]. The presence of the *TP53* missense mutation was correlated with higher tumor-infiltrating CD3+ and CD8+ T-cell densities in primary prostate cancer tissues [83]. A recent study discovered a potential mechanism whereby the speckle-type POZ protein (SPOP) loss-of-function mutations could reduce ubiquitination-mediated PD-L1 degradation, leading to increased PD-L1 levels, accompanied by decreased TILs in both mouse tumors and human primary prostate cancer tissues [84]. Hence, it is imperative to develop and apply integrated genotypic–immunophenotypic analyses in prostate cancer to better understand the underlying molecular features that influence the cancer immunophenotype.

## 4. Strategies for Measuring Prostatic Inflammation

Studies have used a variety of techniques and data sources, both experimental and computational, to estimate the TIME status in past years (Table 2). Most early data sources are based on tissue sections and tissue microarrays. The microscopic examination of H&E-stained slides provide a direct view of immune cells, with primary morphologic information of the cell amount, location, and tissue structure, but it lacks immuno-labeling to identify specific immune cell subsets. Immunohistochemistry and immunofluorescence techniques help to further differentiate immune cell populations based on immunophenotypes, and provide qualitative and quantitative results. These conventional approaches are usually limited to a low number of markers that can be assessed simultaneously. The development of multiplex methods allows more markers to be evaluated on the same tissue section, particularly in the area of immunofluorescence. For instance, a six-color multiplex immunofluorescence panel of CD4, CD8, FOXP3, Ki67, PanCK, and PD-L1 has been applied to assess immune-cell infiltrates in formalin-fixed paraffin-embedded sections from prostate biopsies and radical prostatectomy specimens [85]. With advances in machine learning, the automated digital image analysis presents advantages in the quantification of target objects with high accuracy and reproducibility, compared to the manual examination of a section under a microscope [86]. As an example, TILs could be quantified on images scanned from H&E slides [87], or those processed after immunohistochemistry or immunofluorescence [88,89]. An iterative chromogenic-based multiplex immunohistochemistry has been applied to quantify the densities of eight T-cell phenotypes separately in the tumor epithelial and stromal regions of prostate cancer, using a whole slide image analysis [90]. Since most epidemiological studies have a limited access to sufficient tissue sections for immunohistochemistry or immunofluorescence, especially biopsy specimens with a low volume of tissue, a digital image analysis demonstrates one use for scanned archival H&E images to gain insights into the TIME.

Flow cytometry is another tool that enables a more precise view of the immune cells from not only blood and bone marrow but also solid tissues that can be dissociated into single cells. Various populations can be identified simultaneously using fluorescent-labeled antibodies on each cell from prostate cancer tissues [91,92], though the necessity for fresh prostate specimens may hinder its application within the context of epidemiological studies. Mass cytometry, or CyTOF, is a variant platform from flow cytometry in which antibodies are labeled with heavy metal ion tags and then quantified by time-of-flight mass spectrometry. It allows for up to 40 markers to be measured in a single sample at a rate of 1,000 cells per second. Another advantage of this method is that it could enable the investigation of cell identity and behaviors at the protein level, including posttranslational modifications and proteolysis products, capturing diverse aspects of biological processes [93]. A total of 57 phenotypically-distinct immune cell types were found in the benign human prostate by mass cytometry, with the abundance of specific immune cell clusters varying considerably between patients [94], highlighting the feasibility to phenotype the immune compartment of prostate cancer.

Computational techniques, such as XCell [95], MCPcounter [96], CIBERSORT [97], and TIMER [98], using gene expression data from tissues, can estimate the abundance of the immune infiltrate into the tumor more precisely, and can present more functional characteristics. More recently, next-generation technologies on the basis of high-resolution data contribute much to the improvement of TIME classifications and uncovering immune heterogeneity, which reveals the deeper knowledge of immunotherapy responses and encourages the discovery of new immunotherapy strategies [61]. For example, aiming at characterizing the phenotypic heterogeneity and spatial distribution of target cells, NanoString digital spatial profiling technology, a combination of techniques for the “high-resolution” profiling of immune cells alongside preserving spatial information, was explored. This technology has been used to quantify transcript and protein levels in formalin-fixed tumor specimens from multiple prostate cancer metastatic sites, demonstrating the utility for accurately assessing tumor heterogeneity and identifying aspects of tumor biology involving the immunological composition of metastases [99].

Prospectively, a growing number of studies seeks to better demonstrate the immune landscape in prostate cancer through these new approaches. They have been contributing towards understanding the complex relationship underlying the tumor–immune interaction, and the roles of the hub genes, which may be valuable markers for prognosis prediction, therapy response, and potential therapeutic targets. Immune-related, gene-based novel nomograms and/or signatures have also been identified to assess the cancer risk and outcome [100,101,102,103].

**Table 2:** Main strategies for measuring tissue-based TIME.

Method	Sample Source	Detection Level	Markers Number	Spatial Information	Advanced Analysis Platform
H&E	FFPE	Cellular structure	NA	Yes	HALO, FIJI/ImageJ, QuPath, CellProfiler, Visiopharm
IHC, IF	FFPE	Protein	Up to 60 [104]	Yes	
Flow cytometry	Fresh tissue, FF, FFPE	Protein	Up to 28 [105]	No	viSNE, PhenoGraph, SPADE1, FlowSOM, t-SNE
Mass cytometry	Fresh tissue, FF, FFPE	Protein	Up to 42 [106]	No	
Microarray	Fresh tissue, FF, FFPE	Transcriptomics	High	No	MCP-counter, xCell, TIMER, quanTIseq, EPIC, CIBERSORT
RNA-seq	Fresh tissue, FF, FFPE	Transcriptomics	High	No	
Digital spatial profiling	FFPE, fresh tissue	Protein, transcriptomics	Up to 50 [107]	Yes	NanoString

Abbreviations: FF, fresh frozen; FFPE, formalin-fixed, paraffin-embedded; H&E, Hemotoxylin and Eosin; IF, immunofluorescence; IHC, immunohistochemistry; NA, not available; RNA-seq, RNA-sequencing; TIME, tumor immune microenvironment.

## 5. Clinical Treatment/Lifestyle Intervention Relevant to Inflammation

Mounting evidence for a link between TIME and prostate cancer have led to a number of immuno-oncology clinical trials, which are based on strategies of single agents or synergistic combinations, via various immune approaches, including vaccines, checkpoint inhibitors, adoptive T-cell therapy, and monoclonal antibodies. Men with asymptomatic or minimally symptomatic mCRPC may consider immunotherapy in clinical trials according to the American National Comprehensive Cancer Network (NCCN) guidelines. Sipuleucel-T, a dendritic cell-based vaccine targeting PAP, is the only therapeutic vaccine approved to treat advanced prostate cancer so far, which has presented a promising result in the registration trial. The median survival in the vaccine group was 25.8 months, compared to 21.7 months in the control group, and this treatment constituted a 22% reduction in mortality risk [108]. Pembrolizumab (anti-PD-1) and Ipilimumab (anti-CTLA4) checkpoint blockades, targeting inhibitory molecules at the surface of Tregs, have already been applied for the treatment of several cancer types, but not specifically to prostate cancer. Clinical trials of these treatments in advanced prostate cancer have been carried out. In the nonrandomized phase Ib KEYNOTE-028 trial of Pembrolizumab, 23 patients with advanced prostate cancer were enrolled. Four patients confirmed partial responses and eight patients had a stable disease within this cohort [109]. In a phase III trial of Ipilimumab in the first-line treatment of patients with chemotherapy-naïve mCRPC, no significant difference was found between the Ipilimumab group and the placebo group in terms of overall survival, but an improvement in progression-free survival (median 5.6 vs. 3.8 months; HR 0.67; 95% CI, 0.55 to 0.81) and PSA responses were identified [13]. Recently, Pembrolizumab was supported by the NCCN panel to be used in patients with microsatellite instability-high- or mismatch repair deficiency-mCRPC. It was reported that androgen deprivation therapy could increase both the number of CD8+ T-cells and Tregs, as well as selectively targeting immunosuppressive cell populations, which may be essential for maximizing the immunogenicity of neoadjuvant therapy, providing the potential of combining androgen deprivation therapy with Treg-depleting agents, or a vaccine-based approach in the treatment of prostate cancer [110]. Immunotherapy has gained less therapeutic efficacy in patients with prostate cancer than expected, despite promising advances in other solid tumors, such as melanomas and non-small-cell lung cancer. High tumor PD-L1 expression or alterations in homologous recombination pathway genes, such as CDK12, may be potentially predictive of responses to immune checkpoint inhibitors for prostate cancer patients [111]. Whether there is a role for immunotherapy in prostate cancer, the questions of which patient subgroups are ideal for this approach, and how to improve the treatment strategies and protocols, still require more investigations.

Based on observational study findings, several potentially modifiable lifestyle factors have been tested in randomized controlled trial settings in men with prostate cancer [112], showing effects on systemic and prostate inflammation. Results from exercise trials in men with prostate cancer showed that long-term physical exercise may cause a decrease in circulating levels of proinflammatory cytokines [113], and that acute exercise also influenced serum inflammatory markers and NK cell responses [114,115]. Stress management may also influence inflammation. In a randomized controlled trial conducting web-based cognitive behavioral stress management and health promotion interventions in men with advanced prostate cancer, participants taking both interventions gained decreases in serum IL-10, IL-8, and TNF-α from the baseline to 6 months, although these markers showed a rebound increase from 6 to 12 months [116]. A small study including 29 men with localized prostate cancer who were randomized to a 6-week course of yoga or standard care before radical prostatectomy, showed that patients in the yoga group had decreased numbers of Tregs, myeloid-derived suppressor cells, and a significant reduction in inflammatory cytokine levels, such as G-CSF, MCP-1, and Flt-3 ligands [117]. Furthermore, evidence has indicated diet and certain medications may affect prostate cancer through their interactions with systemic inflammation, though few clinical trials have focused on intraprostatic inflammation. For example, a four-arm Phase II trial, testing combinations of flaxseed and low-fat diets, found that low-fat diets could have effects on plasma levels of NF-κB-regulated inflammatory cytokines and angiogenic factors in men with prostate cancer [118]. Men on active surveillance after a 12-month glucoraphanin-rich broccoli intervention had an attenuated expression of oncogenic pathways, including the inflammatory response [119]. A randomized clinical trial of atorvastatin, prior to radical prostatectomy in 160 statin-naïve prostate cancer patients, suggested that participants with higher-grade disease, randomized to statins, had lower levels of histological tumor inflammation [120]. Overall, while there is evidence that various lifestyle behaviors and interventions affect systemic inflammation, no studies, to our knowledge, have examined prostate inflammation as an endpoint. Moreover, which patient subgroup could benefit most from diet and lifestyle interventions remains to be identified by future studies.

## 6. Conclusions

In conclusion, we provide an insight into the relationship between inflammation and prostate cancer. Current evidence on the microorganism infections and histopathological characteristics of prostatic inflammation were summarized. The clinical and molecular heterogeneity of prostate cancer makes it challenging to predict the relationship of the immune contexture with outcomes. Therefore, characterizing the different immune phenotypes associated with key genomic alterations and subtypes in prostate cancer will form the foundation for understanding the proposed link between inflammation and prostate cancer. Additional work needs to be done to determine the epidemiological association of inflammation with advanced/lethal prostate cancer and to develop strategies for lethal prostate cancer prevention. Sustained investigation is required to establish whether there is a role for immunotherapy in prostate cancer, and there is scope for additional research on identifying patients who may benefit from diet and lifestyle interventions to target inflammation. Future research should aim to understand the immunological cell types, their spatial distribution, and their function within the prostate cancer TIME, in order to uncover new perspectives on prostate carcinogenesis and reveal novel targets for prevention and treatment.

## Figures and Tables

**Table 1:** Impact of lifestyle factors on advanced and lethal prostate cancer outcomes: evidence from recent studies.

Lifestyle Factor	Assessment	HR (95% CI)		Study Design	Author (Year)
		Advanced Prostate Cancer	Lethal Prostate Cancer		
Vigorous exercise	Men in the highest quintile of vigorous activity compared to the lowest quintile	0.70 (0.53–0.92)	0.75 (0.59–0.94)	Prospective cohort	Pernar (2019) [46]
Obesity	Each 5 kg/m^2^ increase in BMI	1.06 (1.01–1.12)	NA	Meta-analysis	Harrison (2020) [57]
		NA	1.13 (1.08–1.20)	Meta-analysis	Jochems (2020) [58]
Inflammatory diet	Each SD increase in inflammatory diet score among men under 65 yrs of age	1.13 (0.99–1.28)	1.16 (1.00–1.35)	Prospective cohort	Fu (2021) [48]
Cigarette smoking	Current smoking compared to never smoked	NA	1.14 (1.05–1.24)	Retrospective cohort	Riviere (2020) [59]
		1.05 (0.87–1.27)	1.27 (0.98–1.65)	Prospective cohort	Rohrmann (2013) [60]
Aspirin	Current aspirin use compared to never used	1.16 (0.96–1.41)	0.80 (0.66–0.96)	Prospective cohort	Downer (2019) [53]
Statin	Current statin use compared to never/past used	0.98 (0.73–1.31)	0.76 (0.60–0.96)	Prospective cohort	Allott (2020) [56]

Abbreviations: BMI, body mass index; CI, confidence interval; HR, hazard ratio; NA, not available; SD, standard deviation.
